# AGA: Interactive pipeline for reproducible gene expression and DNA methylation data analyses

**DOI:** 10.12688/f1000research.6030.2

**Published:** 2015-10-21

**Authors:** Michael Considine, Hilary Parker, Yingying Wei, Xaio Xia, Leslie Cope, Michael Ochs, Elana Fertig

**Affiliations:** 1 Department of Oncology Biostatistics & Bioinformatics, Johns Hopkins University School of Medicine, Baltimore, MD, 21205, USA; 2 Department of Biostatistics, Johns Hopkins Bloomberg School of Public Health, Baltimore, MD, 21205, USA; 3 Department of Statistics and Biostatistics, Rutgers University, New Brunswick, NJ, 08901, USA; 4 Department of Mathematics and Statistics, The College of New Jersey, Ewing Township, NJ, 08618, USA

**Keywords:** automated, genomic, analysis, datasets, DNA, methylation, expression, arrays

## Abstract

Automated Genomics Analysis (AGA) is an interactive program to analyze high-throughput genomic data sets on a variety of platforms. An easy to use, point and click, guided pipeline is implemented to combine, define, and compare datasets, and customize their outputs. In contrast to other automated programs, AGA enables flexible selection of sample groups for comparison from complex sample annotations. Batch correction techniques are also included to further enable the combination of datasets from diverse studies in this comparison. AGA also allows users to save plots, tables and data, and log files containing key portions of the R script run for reproducible analyses. The link between the interface and R supports collaborative research, enabling advanced R users to extend preliminary analyses generated from bioinformatics novices.

## Introduction

While high dimensional genetic data have increased in availability at reduced cost, robust analyses remain labor intensive and costly. Numerous automated software pipelines have been developed in an effort to increase the rate and decrease the costs at which analyses can be completed, including SVAw ^11^, Partek ^4^, InSilicoDB ^18^. Automated Genomics Analysis (AGA) provides a more dynamic experience, allowing the user to start with raw data and a text file containing corresponding sample annotations from either a single or multiple studies. AGA performs all necessary normalization and batch correction, and then enables the user to interactively determine the samples to contrast in the analysis based on the sample annotations. AGA is implemented in R to facilitate adaptation of state-of-the art genomics analysis techniques. Linking R to a web browser-based interface through RStudio’s shiny also facilitates collaborative analyses in research teams with diverse bioinformatics expertise.

AGA bridges the gap between interactive and reproducible analyses for several platforms, including expression arrays, methylation arrays, and processed RNAseq data. Through the interface, the user determines the size and scope of the analyses. AGA first performs data normalization, including the ComBat ^7^ and SVA ^9^ batch correction algorithms to enable comparison across multiple datasets for non-methylation platforms. The software then performs differential analysis ^16^, and gene set analyses ^2, 17^ based upon defined sample groups. Users obtain standard visualization of genomics data, including hierarchical clustering, boxplots and heatmaps as part of the default analysis. Plots and tables summarizing the results from each analysis are customizable through the interface. The figures and tables in AGA are interactive and customizable. In contrast to other point and click software, AGA logs the R code, and exports the workspace with each figure and table, ensuring that each analysis can be reproduced and further customized. The runtime of analyses will depend largely on the desktop hardware, but also on the data platform and optional analyses selected. On a Mac Pro workstation, containing a 3.2 GHz Quad-Core Intel Xeon processor and 10Gb 1066 MHz DDR3 RAM, analyses containing under 100 samples were completed in under 30 minutes.

## Methods

The AGA application is run through R and interactive through web browsers. AGA is implemented with RStudio’s shiny ^13^, integrating the R code used in the analysis with HTML and JavaScript, for the interactive user interface. Usage requires R version 3.0.1 or higher, and either Mozilla Firefox or Google Chrome, and R packages described in the AGA User’s Manual. The program is divided into seven tabs. Clicking the respective Update button generates the results to be displayed in each tab and clicking the Download buttons save the plots and data.

### Data platforms

AGA supports analyses of DNA methylation and gene expression data. Currently, AGA supports DNA methylation analysis on Illumina 450k arrays. It also supports gene expression analysis of any human Affymetrix expression platform, including exon arrays, and normalized gene counts from RNAseq data. Notably, the flexible format for normalized RNAseq data may be adapted to analyze normalized data from other platforms measuring continuous data, many of which we plan to incorporate in future versions of AGA.

### Initiation

Users of AGA select to load annotation files and high throughput genomic data from files in a specified directory. AGA accepts raw CEL files and iDat files for Affymetrix and DNA methylation arrays, respectively where background correction is performed, as well as quantile normalization for the expression arrays. For gene expression microarrays, AGA performs RMA normalization implemented in the Bioconductor package affy ^5^. Probe-level estimates of DNA methylation are computed from iDat files using Illumina standards with the minfi package ^1^. RNAseq data are formatted as individual text files for each sample, assumed to contain gene names and normalized counts for each sample. It is assumed that normalized RNAseq data are formatted as individual text files for each sample, containing gene names and normalized counts for each sample. More details about the format for each data type are provided in the User’s manual. Sample annotations are specified in a CSV file, whose first column matches the names of the data files. By default, it is assumed the annotation file defines the sample batch; however, this can be updated by editing the annotation files to contain a ‘Batch’ column with unique identifiers for each respective batch within the dataset. Further details about the sample annotations are also provided in the User’s manual.

### Sample selection for differential analysis

After loading in the annotation files, AGA users select categories from the annotation for differential expression analysis. AGA automatically groups samples with common levels in each category as groups for differential analysis. Samples may be further subset from the complete dataset from the criteria selected for each group. When selected, AGA updates the display to output the sample size for each group. Samples are set for analysis by clicking the “Run the Analysis!” button. In cases for which samples span multiple batches, the analysis automatically performs ComBat and SVA batch correction protecting for the biological groups in the annotation selected by the user. Help boxes are available to clarify each input field with further details in the User’s manual.

### Interactive plots and tables

The Dendrogram Plot tab in displays unsupervised hierarchical clustering based upon the complete correlation between values of genes (rows) and samples (columns). The Heatmap Plot tab provides an interactive Javascript heatmap of the genomic data, allowing users to customize genes plotted and color rows by sample annotations. For both Dendrograms and Heatmaps, an option is available to view the pre-batch corrected data to show the effects of batch on and efficacy of correction of the data. The Gene Box Plot tab creates boxplots to summarize values of a user-selected gene in the selected groups.

The Differential Results tab displays the results from the differential analysis using empirical Bayes moderated t-statistics with the Bio-conductor Package limma ^16^. Statistics are computed on data that have been batch corrected by combining ComBat with SVA, protecting for the biological groups selected for comparison ^10^. The p-values are adjusted utilizing the Benjamini-Hotchberg method for multiple hypothesis testing ^8^. Optionally, gene set statistics can be performed for each gene set defined in Biocarta and Gene Ontology using a Wilcoxon rank-sum test comparing the t-statistics from the most differentially expressed probe for genes in the set to similarly selected t-statistics for genes outside of the set. If selected, results from gene set analysis are displayed in the GSA Results tab.

## Example

**Figure 1.**
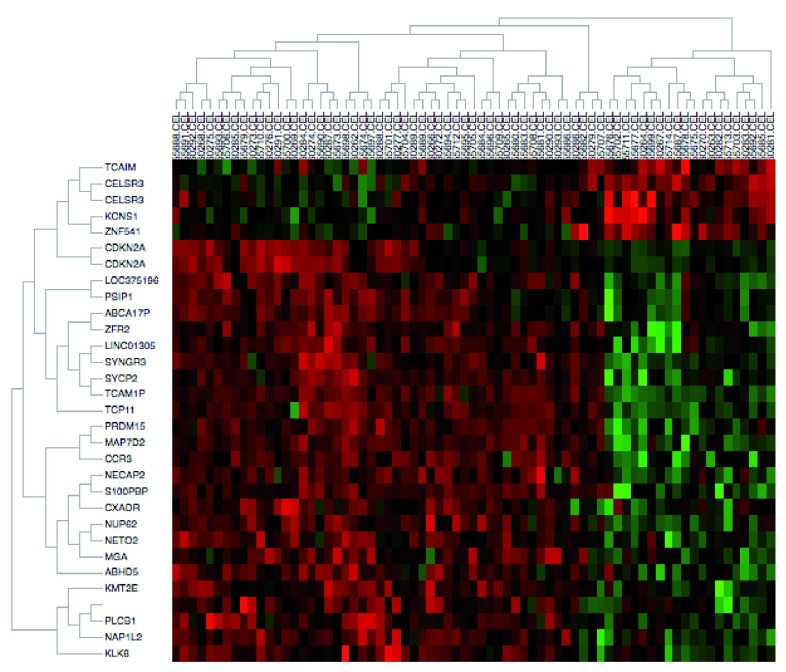
Heatmap displaying the relative expression of the probes with p values below 0.0001 from the example analysis, including CDKN2A. We note that sample names are truncated in the heatmap, but users can reduce the lengths of sample names or ensure that sample identity can be determined by the final characters in the name to associate specific samples with the heatmap.

As an example, we perform analysis on sample datasets containing gene expression of primary head and neck squamous cell carcinoma (HNSCC) tumors. We downloaded measurements from a combination of frozen tumor samples from two distinct studies in GEO available under accession numbers GSE10300 ^3^ and GSE6791 ^12^, representing two distinct batches. Raw CEL files and annotation csv files were obtained as described in the User’s manual. We initialize AGA by selecting the directory containing these data. Once loaded, we check the HPV and Tumor.Source.Type columns to group the samples into primary HPV-positive and HPV-negative tumors for differential expression analysis. We then click “Run the Analysis” to normalize the CEL files with RMA ^6^, batch correct the data with ComBat and SVA, and perform differential expression analysis. The plot in the Dendrogram Plot tab confirms that the batch effects are apparent between these datasets but removed after batch. The heatmap generated in the Heatmap Plot tab ( Figure 1) demonstrates that the batch correction nonetheless preserves gene expression difference between HPV-positive and HPV-negative tumors. Moreover, performing differential expression analysis comparing HPV-positive and HPV-negative HNSCC in the “Differential Analysis” tab confirms the well-established overexpression (p=8.74e-9) of CDKN2A (p16) in HPV-positive HNSCC ^14, 15^.

## Discussion

AGA provides an interface to enable users who may be unfamiliar with R to perform reproducible genomics class comparison analysis. Unlike other automated pipelines, experienced R users can reproduce, extend or modify preliminary analyses. Thus, AGA facilitates collaborations between novice and expert R users for genomics analysis. Future work will extend the AGA pipeline to encode normalization routines to DNA methylation, and analysis routines for other genomics platforms, including copy number data.

## Software availability

### Latest source code

https://gist.github.com/78f566e1a51d745fac3b

### Source code as at the time of publication

https://gist.github.com/F1000Research/9d2acc6aca8ba2d1cc76

### Archived source code as at the time of publication

http://dx.doi.org/10.5281/zenodo.14056 ^19^

### License

GNU GPL V2

## Supplementary material

AGA User’s Manual:
