# Supplementary material for: AGA: Interactive pipeline for reproducible gene expression and DNA methylation data analyses
Source: F1000Res. 2015 Oct 21;4:28. Originally published 2015 Jan 28. [Version 2] doi: 10.12688/f1000research.6030.2 (PMC4617321; doi:10.12688/f1000research.6030.2)
Supplement: Supplementary file 1 [file f1000research-4-7666-s0000.tgz › a7f94f96-9056-4674-a55c-a79d47c0b49b.pdf]

AGA User Manual

Version 1.0

January 2014

Contents

1. Getting Started 3
1a. Minimum Computer Specifications and Requirements 3
1b. Installation........ 3
1c. Running the Application 4
1d. File Preparation........ 4
1e. Example Dataset and Annotation 4
2. Application Use 5
2a. Initialization Tab 5
2b. Dendrogram Tab 10
2c. Heatmap Tab 12
2d. Differential Results Tab 15
2e. Box Plot Tab 17
2f. Gene Set Analysis Tab 18
3. Object Names........ 19
3a. Common:........ 19
3b. Dendrogram: 20
3c. Heatmap: 20
3d. Differential Analysis:........ 20
3e. Gene Set Analysis: 21
3f. Boxplots:........ 21

1. Getting Started

1a. Minimum Computer Specifications and Requirements

A 64-bit operating system

Three Gigabytes of available storage space on primary hard drive

Six Gigabytes of RAM (More required for larger data sets, recommended 12GB+)er

Mozilla Firefox or Google Chrome set as the default internet browser

1b. Installation

Before installing and running the software, Windows users may need to disable their anti-virus software or provide exceptions for the programs being installed.

Download and Install R 3.0.1 (Windows user should install R software onto C:\R-3.0.1, and not the default C:\Program Files\R-3.0.1)

Windows: http://cran.r-project.org/bin/windows/base/old/3.0.1/

Mac: http://cran.us.r-project.org/bin/macosx/old/R-3.0.1.pkg

Download and install RStudio from: http://www.rstudio.com/ide/download/desktop

Windows users may need to install RTools from

http://cran.r-project.org/bin/windows/Rtools/Rtools31.exe

Open RStudio (Windows users may need to "Run as Administrator") and install bioconductor software by copying and pasting these commands into the R console

source("http://bioconductor.org/biocLite.R")

biocLite()

Press Enter to submit the commands. If prompted if you would like to update packages, given the options of "a/s/n" type the letter a and hit enter. In the event that this causes a further issue, such as "cannot replace existing package" or something similar, try again, and input the letter n instead, and hit enter.

Install the shiny package by copying this command into the R console

install.packages('shiny')

Make sure to include quotes around the word shiny or a small error will occur. Select any of the nearby server locations upon the request of the subsequent pop-up window, should one appear.

1c. Running the Application

To access the AGA application, begin with the following commands in the R console

library(shiny); runGist("78f566e1a51d745fac3b")

Your default internet browser should launch at this point. Shiny works best with Google Chrome and Mozilla Firefox, each of which is available for both Mac and PC. It does not work with Internet Explorer, and it is unknown how compatible the application is with Safari.

1d. File Preparation

Format your annotation file to conform to the following standards:

Do not include the use of '#' anywhere within the file

The first column should be the sample file names and left without a column label

Ensure that file names consist of letters, numbers, the dot (period) and/or underscore characters, and start with either a letter or a dot not followed by a number.

If distinct batches are known, insert a column named Batch with that information. Batches with only one sample will be ignored in the analysis.

Save the annotation file as a .csv file, not a .txt or excel spreadsheet. The program only looks for .csv files.

All sample files for a desired analysis should be in the same directory, along with the annotation files.

1e. Example Dataset and Annotation

An example expression dataset can be downloaded from GEO, accession numbers GSE10300 and GSE6791

http://www.ncbi.nlm.nih.gov/geo/download/?acc=GSE10300&format=file

http://www.ncbi.nlm.nih.gov/geo/download/?acc=GSE6791&format=file

Unzip the respective .tar files and then subsequently unzip each of the extracted CEL files (the individual zipped files roughly 5,000KB in size, after they are unzipped, each file will be roughly 13,000KB)

The accompanying example annotation files can be obtained from

https://www.dropbox.com/s/xb92qimvlvj18zg/ChungTest2.csv

https://www.dropbox.com/s/qvq4ad3r5z8jcmd/GSE6791Anno%281%29.csv

REMOVE FILE GSM260280.CEL : it is known to be non-functional (corrupt) and is not included in the analysis. Failure to remove the file will likely cause the running of the analysis to fail.

Again, make sure that all Affymetrix Expression CEL files are in one directory, along with the annotation files.

2. Application Use

2a. Initialization Tab

Begin with replacing the Analysis Title “Automated Genomics Analysis: Head and Neck” with a title for your analysis, this will be used in the naming of saved files, so do not use special characters which may interfere with the saving process- to be safe, stick with letters and numbers.

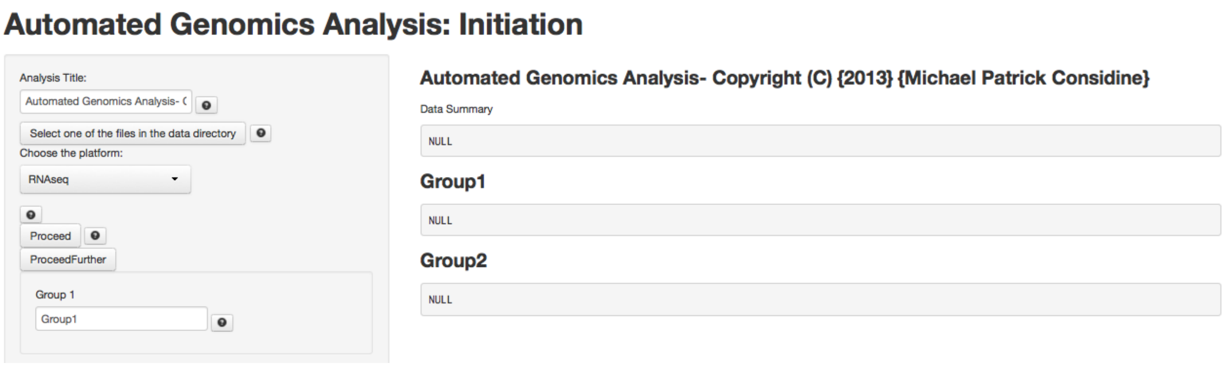

Next, click the “Select one of the files in the data directory” button; if the file selection window does not pop up, open your Rstudio window and look for it there. Navigate to the location in which the annotation files are saved along with the sample files. Select one of the files in the directory. The screen will take you to the R console, so click back to the browser to continue. The data in that directory must be formatted according to the description in the section “File Preparation”.

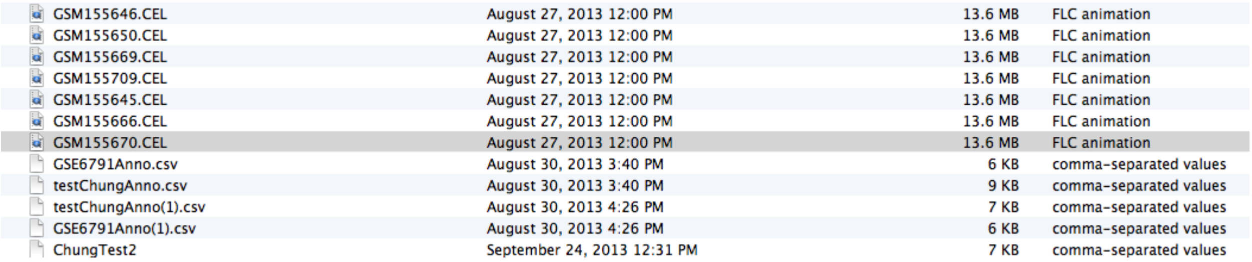

GSM155646.CEL	August 27, 2013 12:00 PM	13.6 MB	FLC animation
GSM155650.CEL	August 27, 2013 12:00 PM	13.6 MB	FLC animation
GSM155669.CEL	August 27, 2013 12:00 PM	13.6 MB	FLC animation
GSM155709.CEL	August 27, 2013 12:00 PM	13.6 MB	FLC animation
GSM155645.CEL	August 27, 2013 12:00 PM	13.6 MB	FLC animation
GSM155666.CEL	August 27, 2013 12:00 PM	13.6 MB	FLC animation
GSM155670.CEL	August 27, 2013 12:00 PM	13.6 MB	FLC animation
GSE6791Anno.csv	August 30, 2013 3:40 PM	6 KB	comma-separated values
testChungAnno.csv	August 30, 2013 3:40 PM	9 KB	comma-separated values
testChungAnno(1).csv	August 30, 2013 4:26 PM	7 KB	comma-separated values
GSE6791Anno(1).csv	August 30, 2013 4:26 PM	6 KB	comma-separated values
ChungTest2	September 24, 2013 12:31 PM	7 KB	comma-separated values

Next select the platform of the data to be analyzed from the drop down menu.

The annotation files you have in the selected directory will appear, along with any other .csv files in the directory. Uncheck any boxes that are not relevant to your desired analysis and press the Proceed button.

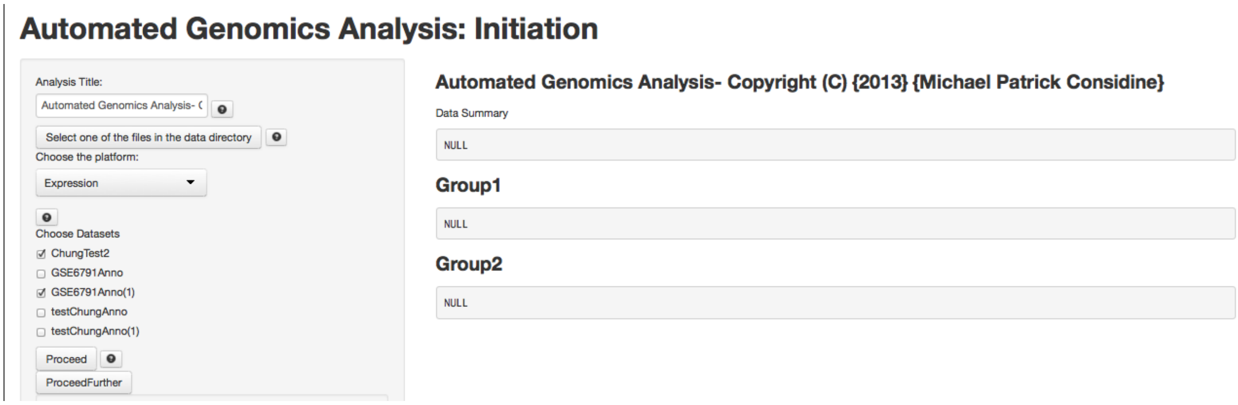

The next step is to determine which columns of the annotation contain the classifiers for which you would like to use in creating your groups to contrast in the analysis. For example, if the desire was to compare HPV positive tumors against HPV negative tumors, the relevant columns could be "HPV status" and "Tumor Status". Following your selection, press the Proceed Further button.

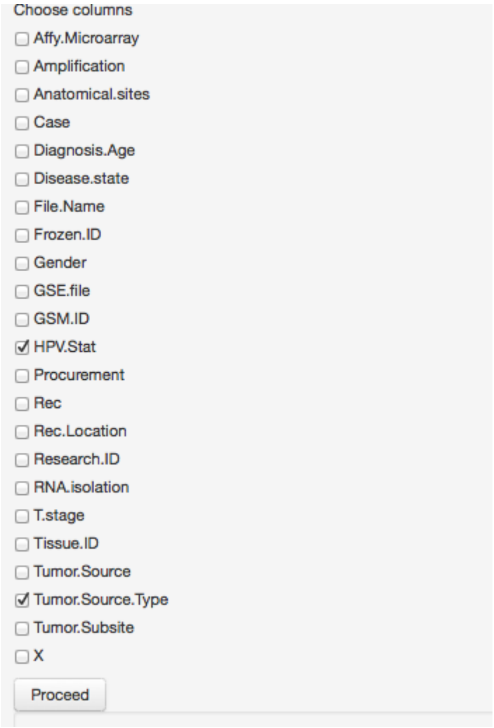

A summary of the information within the columns selected will appear on the right side of the screen.

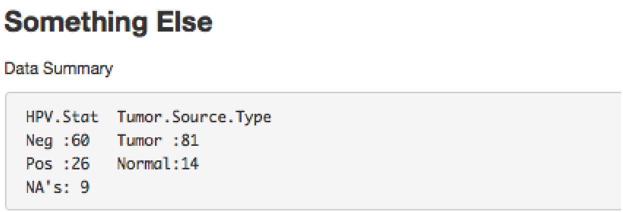

If you wish to rename the groups from Group 1 and Group 2, do so now, otherwise, leave them as Group 1 and Group 2.

The selection of which samples to include in each group is determined by which checkboxes are checked in their respective sections. Continuing the previous example, Group 1 would check the “Positive” checkbox under “HPV status” and “Tumor” under “Tumor status”, while for Group 2, the “Negative” and “Tumor” checkboxes, respectively, would be checked. In other cases, more than one check box per column can be selected if desired. For example, if there were a column for age, and divided the samples into decades, 20’s, 30’s, 40’s, 50’s, 60’s and 70’s, if you wanted to compare samples from those in their 20’s, 30’s, or 40’s to those in their 50’s, 60’s or 70’s, Group 1 could have the first three checkboxes checked, while Group 2 would have the latter three checkboxes checked. The application selects the union of classifiers within a category using the logical OR (samples from patients in their <u>20’s</u>, <u>30’s</u>, or <u>40’s</u>), as well as the intersection between them using the logical AND (samples from <u>tumors</u> of <u>HPV positive</u> patients), and can handle doing both at the same time (samples from <u>HPV positive</u> patients in their <u>20’s</u>, <u>30’s</u> or <u>40’s</u>). After completing this step, press the Proceed! button. On the right side of the screen, the summary of the two groups will appear. If there are samples that would fit into both groups after the subsetting, you will be notified by how many are being excluded from the analysis. In this case, HPV positive tumors are being contrasted with HPV negative tumors.

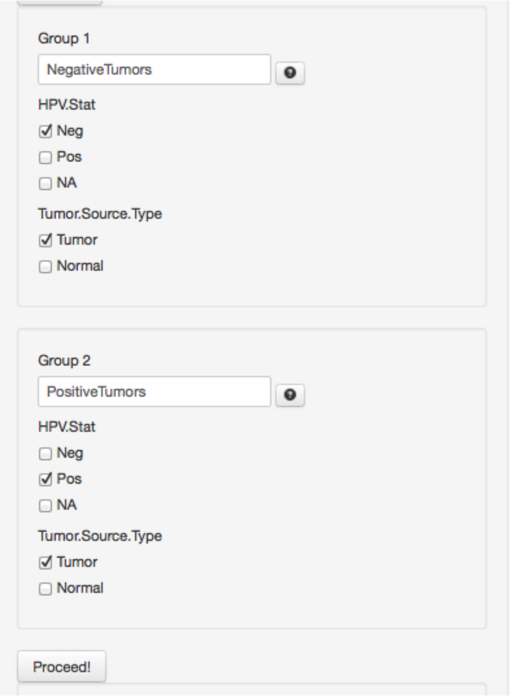

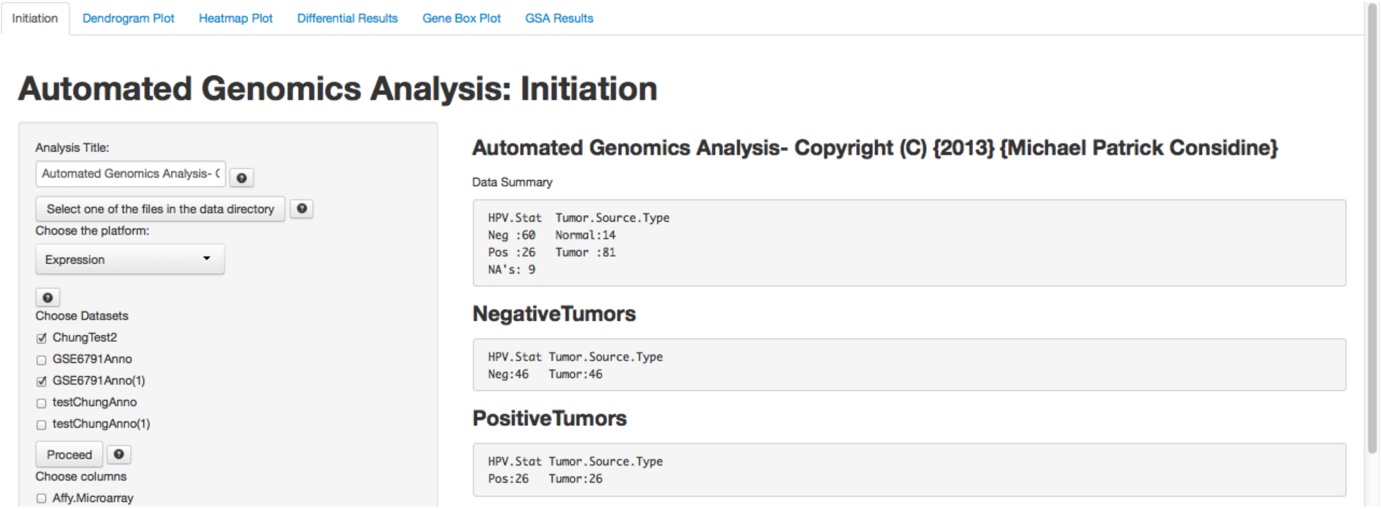
Initiation
Dendrogram Plot
Heatmap Plot
Differential Results
Gene Box Plot
GSA Results
Automated Genomics Analysis: Initiation
Analysis Title:
Automated Genomics Analysis- (
Select one of the files in the data directory
Choose the platform:
Expression
Choose Datasets
ChungTest2
GSE6791Anno
GSE6791Anno(1)
testChungAnno
testChungAnno(1)
Proceed
Choose columns
Affy.Microarray
Automated Genomics Analysis- Copyright (C) {2013} {Michael Patrick Considine}
Data Summary
HPV.Stat Tumor.Source.Type
Neg :60 Normal:14
Pos :26 Tumor :81
NA's: 9
NegativeTumors
HPV.Stat Tumor.Source.Type
Neg:46 Tumor:46
PositiveTumors
HPV.Stat Tumor.Source.Type
Pos:26 Tumor:26

If you would like to run optional additional Gene Set Analyses, check the appropriate boxes. If GSA is selected, checkboxes will appear asking if you would like to look for up regulated, down regulated or mixed gene sets.

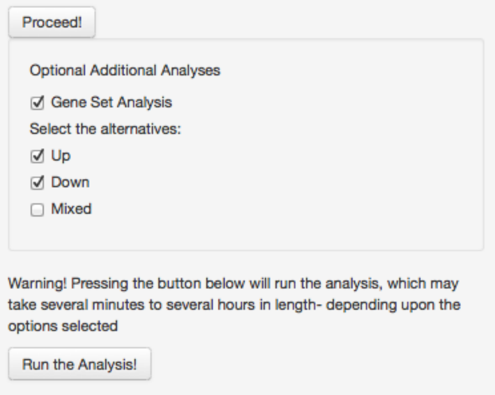

When you are certain that the analysis is set up the way you intended, click the Run the Analysis! button. The length that the application takes to run will vary depending on the number of samples included in the analysis, as well as if optional analyses which were selected to be run. When the analysis is completed (the test run on my machine for this example took 25 minutes), text will display below the summaries on the right side of the screen indicating which tabs you can proceed to. You do not need to follow the tabs in sequential order if you do not desire to do so.

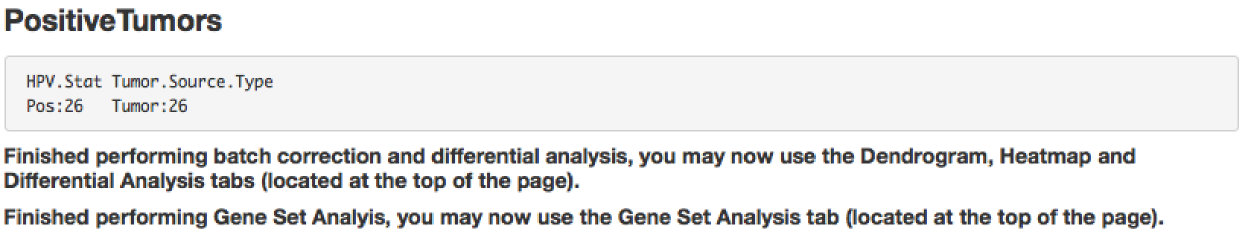

2b. Dendrogram Tab

The first option is to select the column from the annotation to determine the colors of the dendrogram labels. The second option is to select the column to determine the text of the dendrogram labels. The batch column is also available to visualize the removal of batch effects from the data. Click the Update the Dendrogram button to generate the figure.

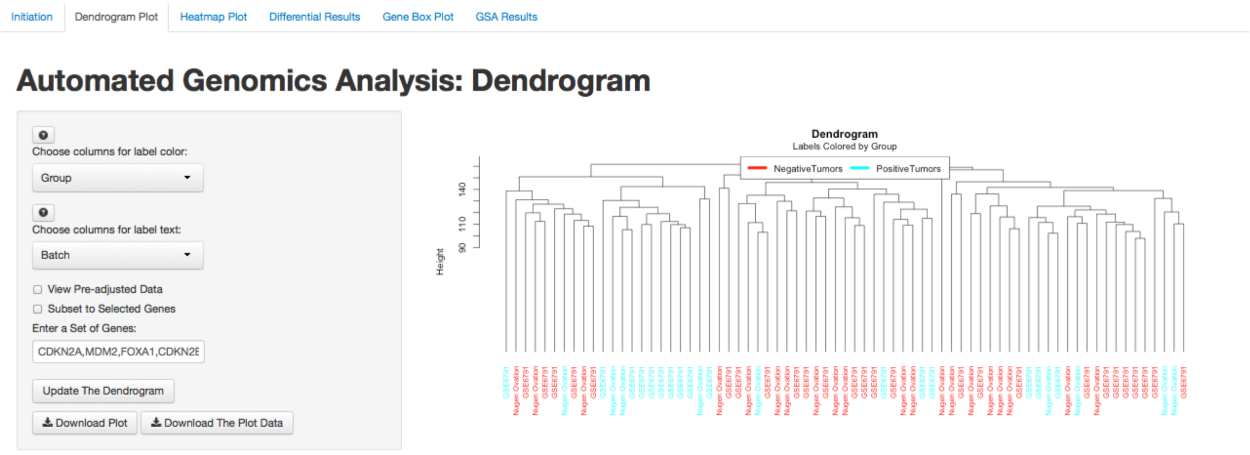

If you wish to view the data before batch correction was utilized as a comparison, check the checkbox and click the Update the Dendrogram button again.

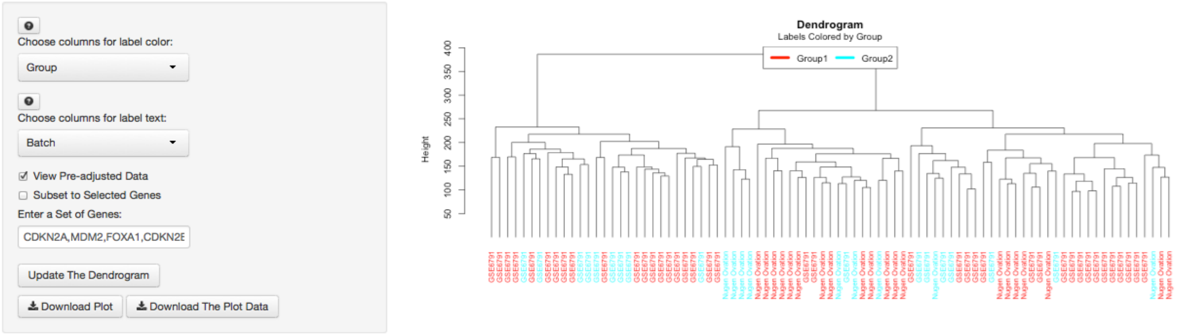

Additionally, you can subset the dendrogram to a custom gene list by entering the gene symbols separated by commas (no spaces, the example format is provided in the field’s default input) and checking the check box ‘Subset to Selected Genes’.

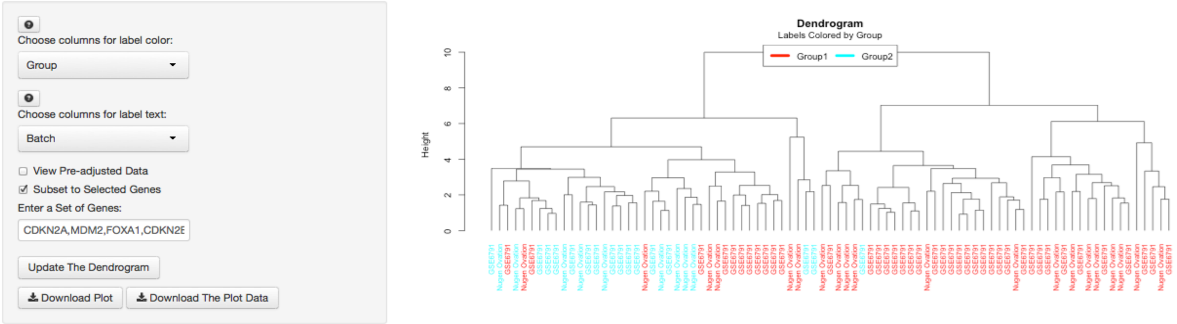

If you wish to download the plot itself to keep, press the Download Plot button. A PDF of the

dendrogram can be saved to a directory of your choice, by default its name will be that which was entered in the initial tab, as well as the time and date of the last pressing of the Update the Dendrogram button.

In order to ensure reproducibility, it is recommended to also download the data used to generate the dendrogram by pressing the Download the Plot Data button. This will save an R data object with the same name as the plot PDF. If the user is familiar with R, they can load the data and continue their analysis outside of the application from this point.

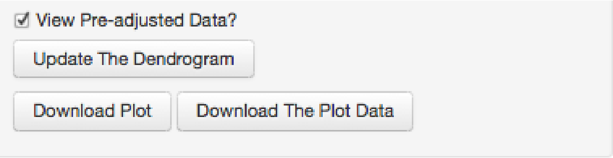

2c. Heatmap Tab

Enter a p value cutoff between 0 and 1. The heatmap will be reduced to probes with an adjusted p value below this threshold.

Enter a fold change cutoff of 0 or higher.

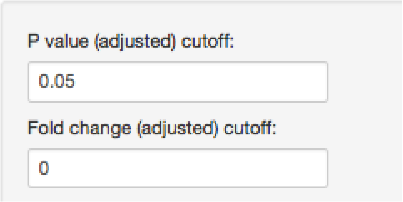

If you wish to subset to gene sets from GO Sets or Reactome Pathways, check the respective box and then start to type part of the set/pathway name. A list of sets from the search will appear in the drop down menu, select the set you wish to subset to.

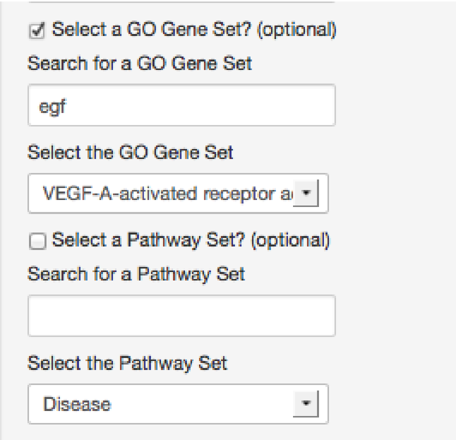

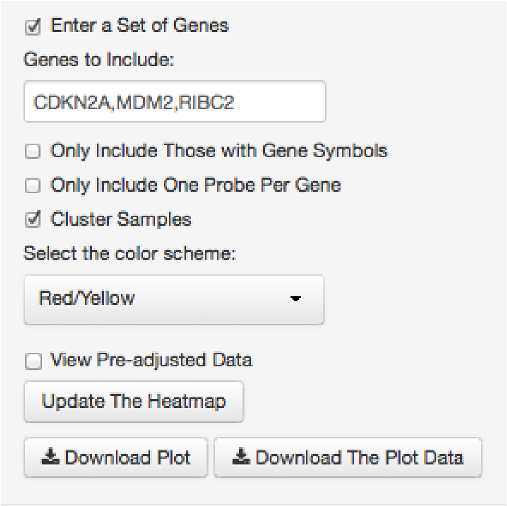

There is an option to show a custom subset of genes, simply enter their gene symbols, separated only by a comma (no spaces!), and it will display only genes in the set provided by the user.

It is not uncommon for probes to not have a gene associated with them, if you wish to remove these from the heatmap, click the relevant checkbox.

Many genes have multiple probes associated with them, if you wish to restrict the heatmap to only the most statistically significant probe for each gene click the associated checkbox.

The heatmap can have the samples clustered via a dendrogram, or not. By default this is turned on. Note that this only applies to the non-interactive heatmap that is produced by the download button, not the interactive heatmap displayed in the browser.

The color scheme of the heatmap is variable; select the colors that you find most to your liking.

There is also an option to view the pre-adjusted data in the heatmap if you would like to do so.

Click the Update the Heatmap button when you wish to generate the heatmap.

The heatmap itself is interactive. The associated sample and gene names will be highlighted when the cursor hovers over a cell in the heatmap. Users can zoom in on the plot by using the mouse scroll wheel while the cursor is hovering over either dendrogram beside the heatmap.

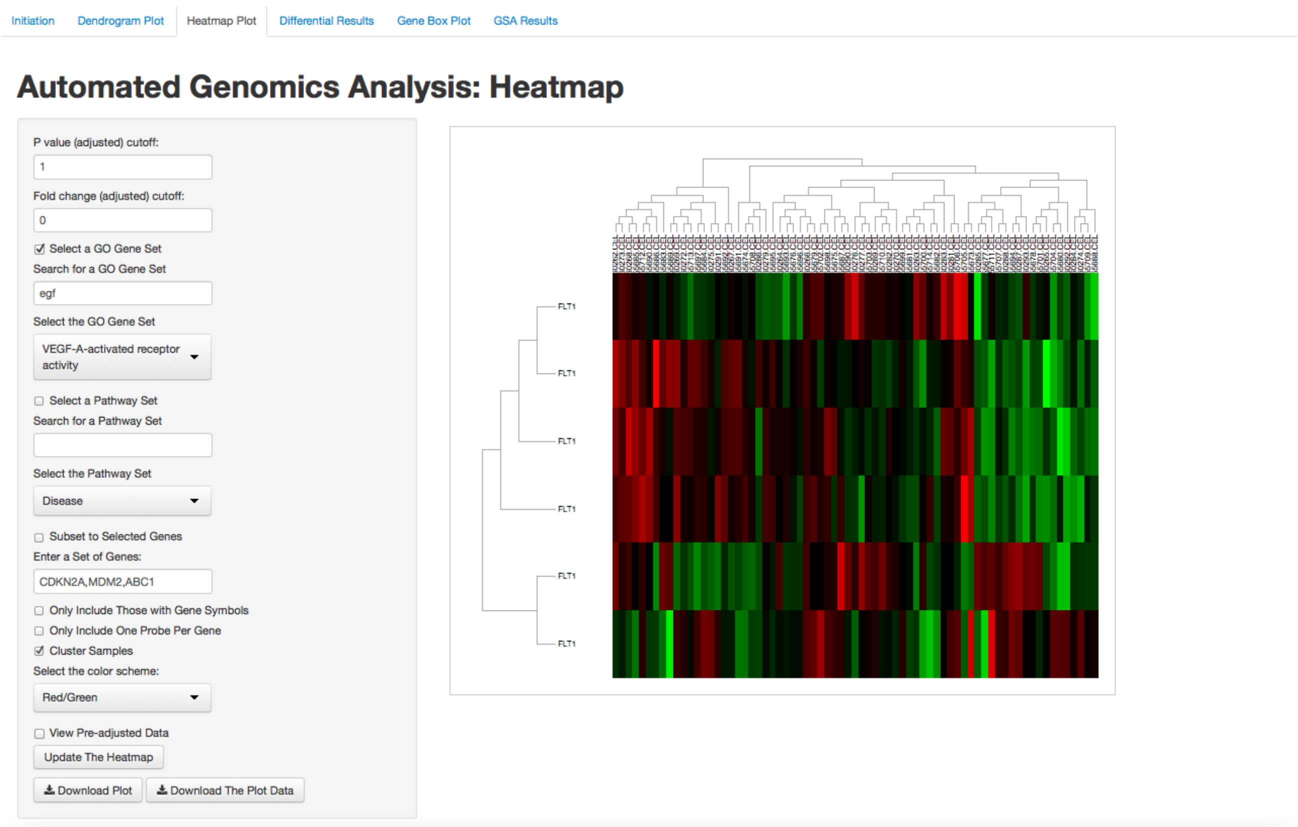

The plot and data can be saved as in the dendrogram. However, the heatmap plot that is saved will mildly differ from that of the interactive heatmap, as different code is utilized to generate each respective heatmap.

2d. Differential Results Tab

The differential results table can be subset in many of the same ways as the heatmap, p value, fold change, gene sets, unique genes and probes associated with genes.

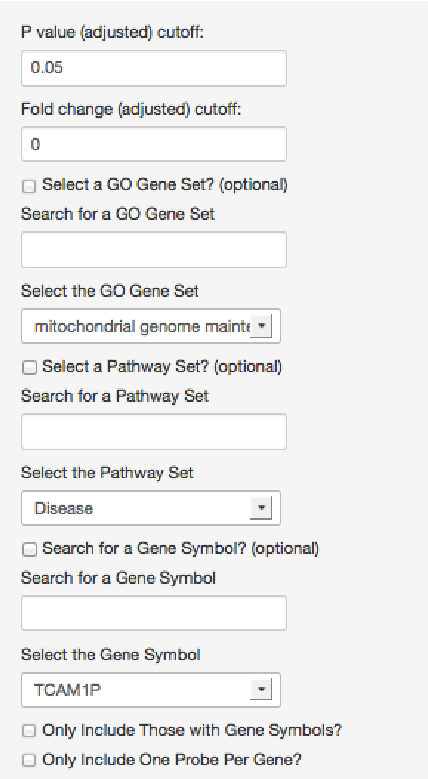

The table can also be subset to a specific gene using a similar method as the gene sets. Also, it is possible to display only up regulated or down regulated genes. With it being Group 1 minus Group 2, up regulated would mean that Group 1 values were higher than Group2, and down regulated would be for results which are lower in Group 1 than they were in Group 2.

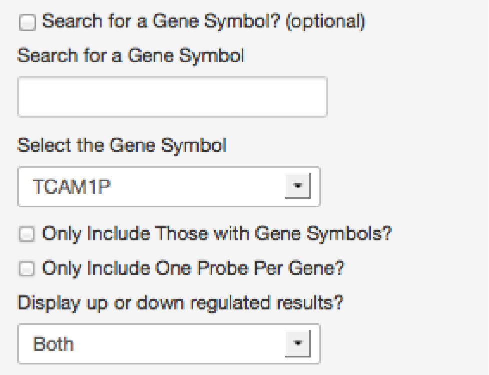

The columns displayed in the table can be reduced to just those you wish to consider.

Choose columns

- ☑ probeSetID
- ☑ sym
- ☑ EntrezID
- ☑ gene
- ☑ logFC
- ☑ AveExpr
- ☑ t
- ☑ B

Update The Table

There is a quick search function that will allow you to quickly look for a specific gene or probe using part of the name. The number of results displayed per page can also be altered. Note that the results saved will not be subset by this search function.

Show 10 entries Search:

Rank	probeSetID	sym	EntrezID	gene	logFC	AveExpr	t	B	P.Value	adj.P.Val
1	233320_at	TCAM1P	146771	testicular cell adhesion molecule 1, pseudogene	1.9853	6.7136	9.2751	20.681	5.7621e-14	3.1504e-9
2	207039_at	CDKN2A	1029	cyclin-dependent kinase inhibitor 2A	3.165	8.1044	8.8776	19.127	3.1961e-13	8.7373e-9
3	233064_at	ZFR2	23217	zinc finger RNA binding protein 2	1.1013	6.758	8.7403	18.588	5.7839e-13	1.0541e-8
4	206546_at	SYCP2	10388	synaptonemal complex protein 2	1.81	5.2933	8.5259	17.744	1.4608e-12	1.9968e-8
5	231164_at	ABCA17P	650655	ATP-binding cassette, sub-family A (ABC1), member 17, pseudogene	1.5851	6.3539	8.1177	16.133	8.5394e-12	9.3379e-8
6	207366_at	KCNS1	3787	potassium voltage-gated channel, delayed-rectifier, subfamily S, member 1	1.1263	7.1437	7.9627	15.52	1.6697e-11	1.5215e-7
7	228262_at	MAP7D2	256714	MAP7 domain containing 2	1.9806	7.3474	7.6009	14.09	7.957e-11	6.215e-7
8	219368_at	NAP1L2	4674	nucleosome assembly protein 1-like 2	-1.6286	6.5118	-7.5168	13.758	1.1434e-10	6.9932e-7
9	205165_at	CELSR3	1951	cadherin, EGF LAG seven-pass G-type receptor 3	0.78227	7.4764	7.5152	13.752	1.1511e-10	6.9932e-7
10	220731_s_at	NECAP2	55707	NECAP endocytosis associated 2	0.37712	9.6478	7.3466	13.087	2.377e-10	0.0000012996

Showing 1 to 10 of 1,000 entries

First Previous 1 2 3 4 5 Next Last

The buttons for Update the Table, Download Table and Download the Table Data function in similar ways to the buttons for the previous plot tabs.

2e. Box Plot Tab

Begin by searching for a gene to select, as in the search/select fields in previous tabs.

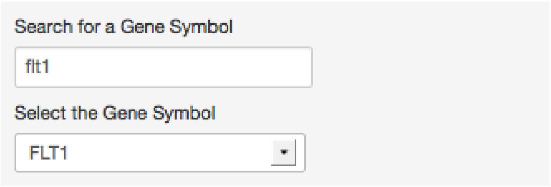

The user can select the colors for each group for the box plots.

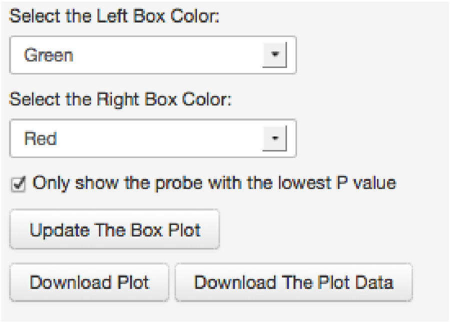

If only one plot is desired, check the box and only the probe for the gene with the lowest p value will appear.

The title of the plot will indicate the gene symbol, probe ID, adjusted p value and the logged fold change between groups.

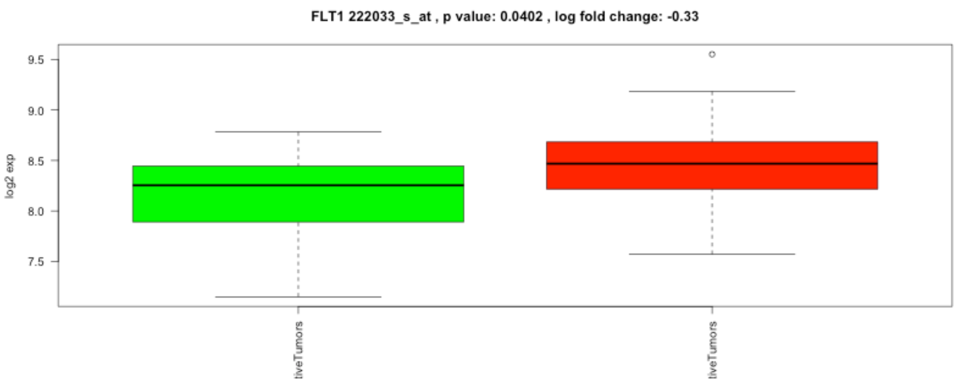

The Update the Box Plot, Download Plot and Download the Plot data function similarly to as in previous tabs, however the plot data will be saved in a zip archive, which will contain all the plot PDF files.

2f. Gene Set Analysis Tab

The p value cutoff between 0 and 1 can be entered, and gene sets can be searched for, and rather than selecting a single set. Any sets that are found to match the search text will be viewable.

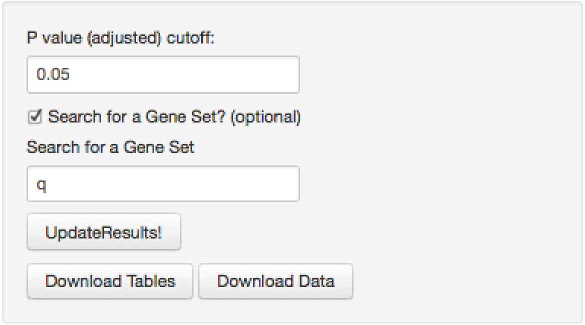

The search will apply to all three categories of gene sets, the Cytoband, Reactome and GO.

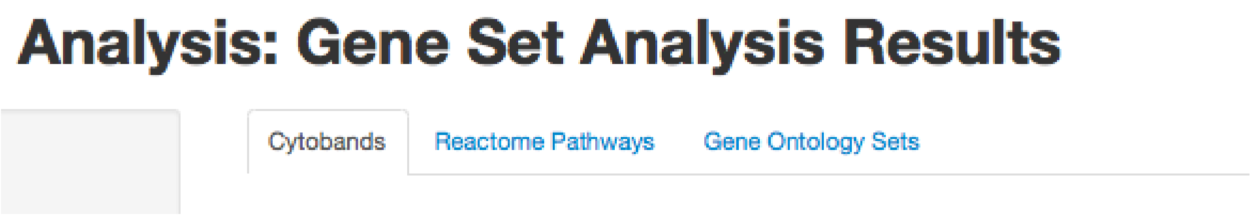

As with the differential results tab, there is a quick search function that will allow you to quickly look for a specific gene or probe using part of the name. The number of results displayed per page can also be altered. Again, the results saved will not be subset by this quick search function.

Up

Show 10 entries   Search: 3q

Rank	Pval	AdjPval	GeneSet
2	4.93006885561768e-09	1.51353113867463e-06	3q29
6	0.000585099548589137	0.0283619307500314	3q13.2
9	0.00107415934706645	0.0449682163021908	3q13.33
10	0.00123317621666588	0.0493806650238817	3q21.1
12	0.00171859495628416	0.0606381703468014	3q22.3
22	0.00424916688378873	0.0850757108689004	3q27
26	0.00618333517231058	0.109516378724962	3q26.33
38	0.0148981918727364	0.181080648863116	3q23
42	0.0163828732514869	0.190995269172397	13q32.1
50	0.0203736649442166	0.2156798323405	3q25.33

Showing 1 to 10 of 58 entries (filtered from 634 total entries)

First Previous 1 2 3 4 5 Next Last

The UpdateResults! Download Tables and Download Data buttons work much as before, but with the tables being saved to a zip archive containing each of the tables generated by the analysis.

3. Object Names

In the .rda files, many objects are supplied, the following is a list of their names and descriptions

3a. Common:

normdat– the normalized and batch corrected data matrix

prenormdat- the data matrix before normalization and batch correction

theg1- name of the group1

theg2- name of the group2

dssub- the list of file names used to generate the annotation

numclasses- the list of the classes used in a vector

sampClasses- list of sample names and their class affiliation

fullAnno- the annotation file in full for the selected samples

3b. Dendrogram:

myhist- the histogram object

isold- true/false, was the use pre-normalized data box checked

labs- plot labels

colr- colors of the labels

3c. Heatmap:

inp- the subset of which probes fit into the filtering from tlist1b

toptab- the full list of probes after differential analysis

tlist1b- the subset of differential analysis results

Colv- the dendrogram, if the columns were clustered

dodendy- true/false if the checkbox for clustering columns was checked

heatcols- the color choice scheme of the heatmap

colr- the color labels for the samples by class

3d. Differential Analysis:

dat.rma- synonymous with normdat

tlist1- all of the differential analysis results

tlist1b- the subset of differential analysis results

topPWselected- the name of the pathway selected for subsetting

topgselected- the name of the gene symbol selected for subsetting

topcols- the columns selected to be displayed

topgenesubbingPW- true/false, is the subsetting to the pathways turned on

topgenesubbing- true/false, is the subsetting to the GO turned on

topgoselected- the name of the GO selected for subsetting

topFCv- value of the fold change cutoff

topsymonlyOne- true/false, is only one probe per gene being displayed

topPval- p value cutoff

topgenesubbingG- true/false, is It subset to a single gene symbol

3e. Gene Set Analysis:

gsaPval- p value cutoff

GSAmix- the results from the mixed analysis before subsetting

GSAup- as above, but for the up analysis

GSAdown- as above, but for the down analysis

GSAmixedx- the results from the mixed analysis after subsetting

GSAupx- as above, but for the up analysis

GSAdownx- as above, but for the down analysis

3f. Boxplots:

subbox- the data subset to the specified gene
