# Supplementary material for: AGA: Interactive pipeline for reproducible gene expression and DNA methylation data analyses
Source: F1000Res. 2015 Oct 21;4:28. Originally published 2015 Jan 28. [Version 2] doi: 10.12688/f1000research.6030.2 (PMC4617321; doi:10.12688/f1000research.6030.2)
Supplement: Peer review history

# Peer review: Referee response for version 1

**DOI:** 10.5256/f1000research.6456.r8835

**Published:** 2015-06-22

**Authors:** Matthew McCall

**Affiliations:** 1 Department of Biostatistics and Computational Biology, University of Rochester, Rochester, NY, USA

The authors describe a software package for interactive (via a shiny webapp) genomic analysis. By running R behind the scenes, this software addresses a common challenge in genomic data analysis -- the transition from simple initial analyses (typically performed by a novice user) and more complex later analyses (typically performed by an advanced user). When the initial analyses are not easily examined / reproduced, the advanced user often must start from scratch. The AGA software will hopefully address this issue. 

The title of the article is currently too broad -- the software is only able to handle Affymetrix expression arrays, Illumina 450k methylation arrays, and normalized RNA-seq gene counts. However, I trust that the authors will expand the functionality of the software to handle many other platforms and types of genomic data. 

My primary criticism of this work is that I was unable to successfully use the package. The package depends on a large number of other packages (shinyIncubator, googleVis, and heatmap among others). In particular, I was unable to install the heatmap package. Additional instructions on how to obtain / install all of the required dependencies should be added to the user manual.

I also have a few minor criticisms of the article:

1. In Figure 1, the gene names are not legible due to over-plotting, and the column names are truncated.
2. The citation for the fRMA method is incorrect. The correct citation is:
  McCall MN, Bolstad BM, and Irizarry RA (2010). Frozen Robust Multi-Array Analysis (fRMA), Biostatistics, 11(2):242-253.

I have read this submission. I believe that I have an appropriate level of expertise to confirm that it is of an acceptable scientific standard.

# Response

**Published:** 2015-09-18

**Authors:** Michael Considine

**Affiliations:** Johns Hopkins University, USA

1) *The title of the article is currently too broad -- the software is only able to handle Affymetrix expression arrays, Illumina 450k methylation arrays, and normalized RNA-seq gene counts. However, I trust that the authors will expand the functionality of the software to handle many other platforms and types of genomic data.*

The title has been revised to "AGA: Interactive pipeline for reproducible gene expression and DNA methylation data analyses"

2) *My primary criticism of this work is that I was unable to successfully use the package. The package depends on a large number of other packages (shinyIncubator, googleVis, and heatmap among others). In particular, I was unable to install the heatmap package. Additional instructions on how to obtain / install all of the required dependencies should be added to the user manual.*

The number of packages is necessary for the diverse functionality and formatting, and to make the best possible application.  We have added instructions to manually install the required packages on page 4 of the revised user’s manual.

“In the event of difficulties installing libraries in R, copy lines 144 to 200 in the global.r script and enter them into your R console.  Afterwards, run the command: sporelibs()”

*I also have a few minor criticisms of the article:*

*3) In Figure 1, the gene names are not legible due to over-plotting, and the column names are truncated*

We have revised Figure 1 to reduce the number of genes so that the gene names are legible. The column names are set by the sample identifiers and must be truncated to facilitate visualization. We note in the revised caption to Figure 1 that: “We note that sample names are truncated in the heatmap, but users can reduce the lengths of sample names or ensure that sample identity can be determined by the final characters in the name to associate specific samples with the heatmap”.

4) *The citation for the fRMA method is incorrect. The correct citation is:*

By default, AGA implements RMA instead of fRMA. We have removed this citation from the revised manuscript and clarified this choice in by adding this second sentence of Initiation subsection of the revised manuscript: “For gene expression microarrays, AGA performs RMA normalization implemented in the Bioconductor package affy” and revising the fourth sentence of the Example subsection of the revised manuscript “We then click “Run the Analysis” to normalize the CEL files with RMA”

# Peer review: Referee response for version 1

**DOI:** 10.5256/f1000research.6456.r7514

**Published:** 2015-02-02

**Authors:** Subha Madhavan

**Affiliations:** 1 Innovation Center for Biomedical Informatics, Georgetown University, Washington, DC, USA

The authors have described Automated Genomics Analysis (AGA), an interactive program to analyze high-throughput genomic data sets on a variety of platforms.

The software is implemented in R with web app using Shiny.

Specific comments are noted below:

1. cBIOPortal is listed as an example for reducing cost of genomic analysis using AGA. cBioPortal's purpose is to help researchers mine analyzed results and it is available for free for non-commercial use. cBIOPortal is not in the same class of software as AGA and is an inappropriate comparison.
2. Title needs to be changed - current AGA software supports expression and methylation analysis only. The title is very broad, especially given that there is not support for genomic variant analysis in the software.
3. Describe any quality checks performed on CEL and idat files briefly beyond batch correction. How does the software deal with missing values?
4. Address scalability. How does the software scale for large studies?

I have read this submission. I believe that I have an appropriate level of expertise to confirm that it is of an acceptable scientific standard.

# Response

**Published:** 2015-09-18

**Authors:** Michael Considine

**Affiliations:** Johns Hopkins University, USA

1) *cBIOPortal is listed as an example for reducing cost of genomic analysis using AGA. cBioPortal's purpose is to help researchers mine analyzed results and it is available for free for non-commercial use. cBIOPortal is not in the same class of software as AGA and is an inappropriate comparison.*

We have removed reference to cBIOPortal in the revised manuscript.

2) *Title needs to be changed - current AGA software supports expression and methylation analysis only. The title is very broad, especially given that there is not support for genomic variant analysis in the software.*

The title has been revised to "AGA: Interactive pipeline for reproducible gene expression and DNA methylation data analyses"

3) *Describe any quality checks performed on CEL and idat files briefly beyond batch correction. How does the software deal with missing values?*

We have revised the Initiation subsection of the Methods section to note that: “For gene expression microarrays, AGA performs RMA normalization implemented in the Bioconductor package affy ^5^.  Probe-level estimates of DNA methylation are computed from iDat files using Illumina standards with the minfi package ^1^. RNAseq data are formatted as individual text files for each sample, assumed to contain gene names and normalized counts for each sample.

4) *Address scalability. How does the software scale for large studies?*

We have revised the Introduction section to include more information on the length of analyses, “The runtime of analyses will depend largely on the desktop hardware, but also on the data platform and optional analyses selected.  On a Mac Pro workstation, containing a 3.2 GHz Quad-Core Intel Xeon processor and 10Gb 1066 MHz DDR3 RAM, analyses containing under 100 samples were completed in under 30 minutes.”
